# Building research capacity among health care professionals in LMICs: experiences from the 9th Tata Annual Biostatistics and Research Methods Course, 2025

**DOI:** 10.3332/ecancer.2026.2077

**Published:** 2026-02-19

**Authors:** Soumitra Shankar Datta, Jigeesha Ghosh, Dishari Choudhury, Suvro Sankha Datta, Prateek Jain, Sakshi Adhia, Indranil Mallick, Sanjit Agrawal, Sanjay Garg, Shouriyo Ghosh, Arijit Nag, Arnab Mukherjee, Soumita Ghose, Sayantani Das, Rima Mukherjee, Sujit Sarkhel, Pattatheyil Arun

**Affiliations:** 1 Department of Palliative Care & Psycho-oncology, Tata Medical Center, New Town Rajarhat, Kolkata 700160, India; 2 Institute of Clinical Trials and Methodology, University College London, 90 High Holborn, London WC1V 6LJ, UK; 3 Department of Transfusion Medicine, Tata Medical Center, New Town Rajarhat, Kolkata 700160, India; 4 Department of Head and Neck Surgery, Tata Medical Center, New Town Rajarhat, Kolkata 700160, India; 5 Department of Radiation Oncology, Tata Medical Center, New Town Rajarhat, Kolkata 700160, India; 6 Department of Breast Surgery, Tata Medical Center, New Town Rajarhat, Kolkata 700160, India; 7 Fortis Hospital, 730, Eastern Metropolitan Bypass, Anandapur, East Kolkata Twp, Kolkata, West Bengal 700107, India; 8 Indian Psychiatric Society - West Bengal Chapter, Dr BC Roy IMA House, 11/3 Dr Biresh Guha Street, Kolkata, Circus Avenue, Kolkata, West Bengal 700017, India; 9 Department of Clinical Haematology and Cellular Therapies, Tata Medical Centre, New Town Rajarhat, Kolkata 700160, India; 10 Department of Administration and Policy, Tata Medical Center, Major Arterial Road, New Town, Rajarhat, Kolkata 700160, India; 11 Department of Psychiatry, Woodlands Multispeciality Hospital, 8/5, Alipore Road, Kolkata 700 027, India; 12 Editor in Chief, Indian Journal of Psychiatry, Department of Psychiatry, IPGMER and SSKM Hospital, 7, D. L. Khan Road, Kolkata, West Bengal 700025, India; 13 Chief Executive Officer, Tata Medical Center, Major Arterial Road, New Town, Rajarhat, Kolkata 700160, India; a https://orcid.org/0000-0003-1674-5093; b https://orcid.org/0009-0002-5666-3447; c https://orcid.org/0009-0002-2284-8945; d https://orcid.org/0000-0003-2094-6429; e https://orcid.org/0000-0001-5077-0749; f https://orcid.org/0000-0002-5567-9204; g https://orcid.org/0000-0003-2804-7815; h https://orcid.org/0000-0002-6325-7116; i https://orcid.org/0000-0003-1674-5093; j https://orcid.org/0009-0000-2424-2715; k https://orcid.org/0000-0002-6646-7469

**Keywords:** cancer, statistics, research methods, training, LMICs, health care staff, doctors, oncology

## Abstract

Modern medicine, especially oncology in low- and middle-income countries (LMICs), requires clinicians to remain updated in a rapidly evolving field of medicine in the face of a high clinical load. Clinicians need to be able to critically evaluate published evidence and make informed decisions about the individual patients they treat. A clinical culture that encourages clinicians to question and think critically would produce high-quality research from parts of the world that have highest disease burden but lowest contribution to published research. A two-day research methods course was conducted jointly by the Tata Medical Center, Kolkata and the West Bengal Chapter of the Indian Psychiatric Society on 22nd–23rd August 2025. We report on our experience of organising this course and the lessons learned from interacting with the audience in an LMIC setting.

Live anonymous participant responses were captured using Mentimeter software during the training, and written anonymous feedback were provided by majority of attendees. The three main barriers to conducting research that our participants reported were: ‘lack of training in research’, ‘difficulties in writing a research paper’ and the ‘researcher’s personal circumstances’. The participants in our course comprised both men and women clinicians, mostly in their early careers and this group of learners appreciated hands-on training on literature search, reference management and working with the SPSS statistical software to conduct standard statistical tests. To achieve this, institutions and individuals need to foster a conducive environment for research, inspiring those who will be responsible for the future health care delivery.

## Introduction

While practising medicine, especially oncology, clinicians need to have a good grasp of the concept of ‘risk’ [1] as clinical decisions have to be weighed in terms of potential benefit and harm [2]. To do so, they must develop a critical approach to thinking early in their career that enables them not only to evaluate published medical literature but also to determine whether the evidence presented can be applied to the specific context in which they carry out their clinical practice. There has been an exponential growth in published medical literature; from ten medical journals at the end of the 17th century, the number increased to 100,000 by the end of the 20th century [3]. Clinicians in all sub-specialities of medicine need to be capable of assimilating and understanding the scientific basis of medical evidence. Much of the learning can be done across multiple specialities. Over the past decade, as we developed research methods training in oncology, we reflected on the available literature on teaching medical statistics and research methods. A recent review on integrating ‘research’ and ‘teaching’ reported the challenges of incorporating teaching research methods into higher education curricula as follows: a) limitation of resources, b) difficulties in balancing ‘training in research methods’ and the ‘core curriculum’ and c) absence of ‘research-oriented institutional culture’ [4]. They suggested that successful strategies could include timely curricular updates, encouraging faculty involvement in research, cross-disciplinary collaboration and the horizontal and vertical integration of research, starting with the undergraduate curriculum and extending forward [4]. In order to encourage multi-disciplinary learning and discussion, the 9th Tata Annual Biostatistics and Research Methods Course 2025 was jointly organised by the Tata Medical Center, Kolkata, a state-of-the-art cancer centre in Eastern India and the West Bengal Chapter of the Indian Psychiatric Society, a national association of psychiatrists from all over India. As this programme was a continuation of an ongoing initiative, the 2-day programme was designed based on existing expertise and feedback from participants from previous years. The lectures incorporated straightforward examples from oncology, enabling participants to comprehend research methodologies easily. This approach was consistent with the tradition of joint events organised by ecancer Global Foundation and Tata Medical Center, Kolkata, over the past decade. [5, 6].

### Programme details

The 9th Tata Annual Biostatistics and Research Methods Course 2025 was conducted over 2 days, with participants from various parts of the country in attendance. All delegates were required to pre-register online at least 1 month in advance, after paying the applicable course fees. The course included didactic lectures on specific topics related to ‘research methods’, selective tests used in ‘biostatistics’ and interactive workshops involving multiple faculties.

Dr. Soumitra S Datta, organising chair for the course, introduced the course by delivering the welcome address, where he highlighted the importance of learning research methodology and biostatistics in the field of medicine and other allied health fields. The welcome address also included a summary of the demographic information and professional backgrounds of all registered participants, which showed that, in addition to doctors, nurses, psychologists, medical/healthcare administrators, and other professionals also participated in the course. This was followed by a welcome address from Dr. P Arun, Chief Executive Officer of Tata Medical Center Trust, Kolkata, who acknowledged the importance of research. Dr Aniruddha Deb and Dr Sanjay Garg welcomed the delegates on behalf of the West Bengal Chapter of the Indian Psychiatric Society. They spoke briefly on the importance of research in clinical practice. The overview of the 9th Tata Annual Biostatistics and Research Methods Course 2025 is captured in Video 1.

An application called ‘Mentimeter’ was used to gather free-text responses from the participants, thereby engaging the audience. A series of questions were displayed throughout the course for the participants to respond to. Participants could join the quiz online by scanning a QR code. Free-text responses collected from the Mentimeter were analysed using the principles of thematic analysis used in qualitative research.

**Figure 1.**
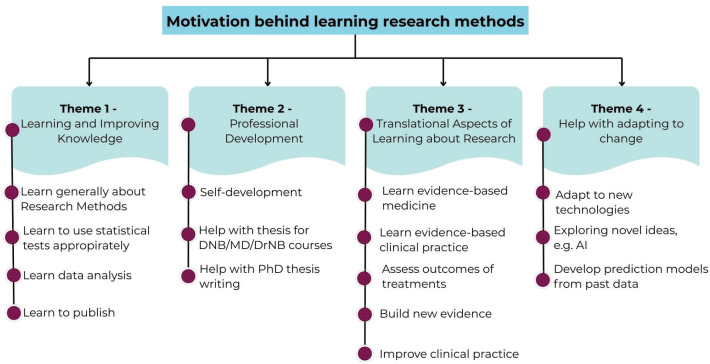
Synthesis of free text responses on motivation to participate in the research methods course.

One of the first questions asked was ‘Why do you want to learn research methods?’ The question was open-ended and each participant could give up to three responses. The main themes and sub-themes which emerged from the answers to this question are presented in Figure 1. The four themes that emerged on the underlying motivation to attend the research methods course are: a) learning and improving knowledge on research methods (‘improve academic knowledge’, ‘strategic learning’, ‘improve rigour’ and so on) b) professional development (‘self development’, help with DNB/MD/DrNB thesis and so on) c) Translational aspects of learning (‘learn about Evidence Based Medicine’, ‘assess outcomes of treatments’, ‘contribute to building new evidence’, improve clinical practice and so on) d) Help with adapting to change (‘adapt to new technologies’, ‘develop prediction models from past data’).

From Figure 1, it is evident that the participants were motivated to learn about research methods to achieve their goals of gaining knowledge about research methodology and statistical analysis, developing their professional skills, improving clinical outcomes and making an impact on their own work in their respective roles.

The 2-day Biostatistics & Clinical Research Methodology course was designed to transition from the basics of biostatistics and research methods to more complex skills, such as learning to use the SPSS software to conduct standard statistical tests on a hypothetical dataset. The audience was provided an opportunity to ask questions at the end of each session.

#### Biostatistics and research methods course (9:00–17:30) - day 1 (22nd August 2025)

The first day of the Biostatistics & Clinical Research Methodology Course consisted of six technical sessions spanning over 8 hours covering a wide range of topics, including the basics of statistics, data analysis, research ethics and how to write a paper. Additionally, a workshop on PubMed search and Reference Management using Zotero was held.

### Technical session 1: types of data/measurement scales/measurements of central tendency/dispersion/sample size calculation

This session was conducted by Dr Arijit Nag, Senior Consultant in the Department of Clinical Haematology at Tata Medical Center, Kolkata. Dr. Nag explained the basics of biostatistics in a simplified manner in his presentation. He set the tone for the upcoming sessions by covering topics such as grouped and ungrouped data, measures of central central tendency and ways to calculate sample size.

### Technical session 2: bivariate statistics (Chi-square, Fisher's Exact test, Student's t-test, Mann-Whitney test)

Dr Indranil Mallick, Senior Consultant, Department of Radiation Oncology, Tata Medical Center, Kolkata, conducted two sessions, one after the other. The first session was on Chi-square, Fisher's exact test, Student's *t*-test and the Mann-Whitney test.

### Technical session 3: tests of significance (p-value and confidence interval)

Dr Mallick continued his second session, where he covered *p*-value and confidence interval and discussed data analysis. The two sessions by Dr Mallick were very interactive and used universally understandable examples. Video 2 by Dr Mallick showcases the journey of a clinician-researcher over time.

### Technical session 4: writing a research paper (IMRAD)

Dr Soumitra S Datta, Senior Consultant Psychiatrist, Tata Medical Center, Kolkata and organising chair for the course, conducted a session on writing a research paper, covering the main aspects of Introduction, Methods, Results and Discussion (IMRAD). His presentation explained the basics of introducing research problems, presenting research findings and writing the discussion section of a research paper. Dr Datta also employed the same free-text software ‘Mentimeter’ to engage the audience with a few multiple-choice questions. These responses were quantitatively analysed based on percentage.

**Figure 2.**
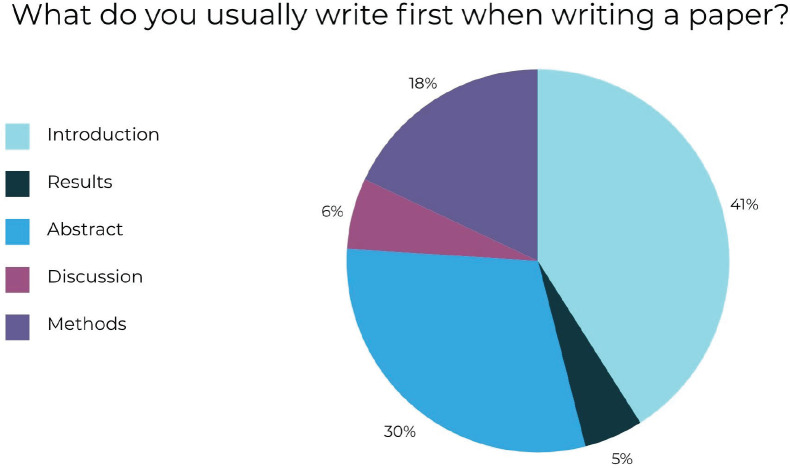
Percentage of responses to ‘What do you usually write first when writing a paper?’

The first quantitative question was, ‘What do you usually write first when writing a research paper?’ and the options that the participants could choose from to respond were Introduction, Results, Abstract, Discussions or Methods. Sixty-one participants responded to this question, of which 25 (41%) participants responded to ‘Introduction’, 18 (30%) participants answered ‘Abstract’, 11 (18%) participants responded to ‘Methods’, 4 (6%) participants answered ‘Discussions’ and 3 (5%) participants responded to ‘Results’. These results have been summarised using the following pie chart (Figure 2).

**Figure 3.**
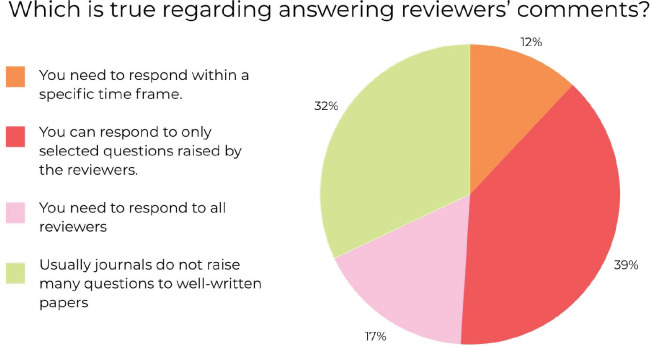
Percentage of responses to ‘Which is true regarding answering reviewers' comments?’

The final question in this segment was ‘Which is true regarding answering reviewers' comments?’ and 59 participants answered it. Of the 59 participants who responded, 23 (39%) participants chose ‘You can respond to only selected questions raised by the reviewers’, 19 (32%) participants chose ‘Usually journals do not raise much questions to well written papers’, 10 (17%) participants chose ‘You need to respond to all reviewers’ and 7(12%) participants chose ‘You need to respond within a specific time frame’. These results have been summarised using the following pie chart (Figure 3).

### Technical session 5: diagnostic accuracy testing (sensitivity, specificity, predictive power, ROC curve)

This session was conducted by Dr. Suvro Sankha Datta, Senior Consultant in the Department of Transfusion Medicine, Tata Medical Center, Kolkata and Joint Organising Secretary for the course. Dr. Datta covered the concepts of sensitivity, specificity, predictive power and ROC curve with relevant examples.

### Technical session 6: research ethics

Dr Prateek Jain, Senior Consultant in the Department of Head and Neck Surgery, Tata Medical Center, Kolkata and Joint Organising Secretary for the course, conducted an interesting session on research ethics. He discussed the principles of non-maleficence, beneficence, justice and autonomy, engaging the audience in an interactive and participatory discussion.

### Workshop 1: PubMed literature search and reference management (Zotero)

Dr Prateek Jain & Dr Shouriyo Ghosh (Associate Consultant, Clinical Haematology and Cellular Therapies, Tata Medical Center, Kolkata) conducted the workshop together. Dr. Ghosh was the first speaker and he covered PubMed literature search. Dr. Jain facilitated the session on using Zotero for reference and citation management. Both workshop facilitators demonstrated the software while simultaneously engaging with and assisting the audience in using it.

**Figure 4.**
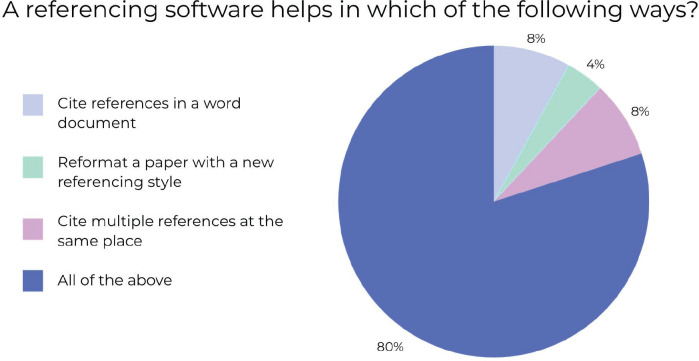
Percentage of responses to ‘A referencing software helps in which of the following ways?’

Mentimeter was employed to ask the audience, ‘A referencing software helps in the following ways?’. The options that the participants could choose from to respond were ‘Cite references in a Word document’, ‘Reformat a paper with a new referencing style’, ‘Cite multiple references at the same place’ or ‘All of the above’. A total of 60 participants responded to this question of which 5 (8%) participants chose ‘Cite references in a word document’, 2 (4%) participants chose ‘Reformat a paper with a new referencing style’, 5 (8%) participants chose ‘Cite multiple references at the same place’ and 48 (80%) chose ‘All of the above.’ The fact that the majority of participants chose ‘All of the above’ suggests that a research methodology course that has a specific workshop on reference management is vital for fulfilling the learning goals of young clinicians. These results have been summarised using the following pie chart (Figure 4).

#### Biostatistics and research methods course (9:00–17:30) - day 2 (23rd August 2025)

The second day of the Biostatistics & Clinical Research Methodology Course consisted of three technical sessions covering more specific and complex topics related to data analysis and research, as well as a workshop on using SPSS.

### Technical session 7: RCT and systematic reviews

Dr Soumitra S Datta began the day’s proceedings with a session on Randomised Control Trial (RCT) and Systematic Review. He covered the basics of RCT with the research design and relevant concepts, along with examples from previous relevant research. He then moved on to discussing Systematic Review.

### Technical session 8: survival analysis- an overview

This session was conducted by Dr Sanjit Agrawal, Senior Consultant, Department of Breast Surgery, Tata Medical Centre, Kolkata. He discussed the basics of survival analysis. Dr Sanjit also addressed the application of survival analysis in oncology.

### Technical session 9: correlation and regression

Dr Soumitra S Datta conducted this session. He began by recapitulating the basics of statistics that had been discussed on Day 1, and then proceeded to explain the concepts of correlation and regression analysis. He tried to simplify complex ideas for the participants to grasp. The participants engaged in interaction with the resource person and asked many questions.

### Workshop 2: SPPS hands-on training

The second workshop for the course was jointly facilitated by Dr Sanjit Agrawal & Dr Soumitra S Datta. The participants had already downloaded a trial version of SPSS, and a data set had been shared with them prior to the workshop so that they would be better prepared. Dr Agrawal began by explaining the basics of entering data into the SPSS software, setting the data values and then explained the method of generating results. Over the next few hours, he explained how to calculate descriptive statistics and conduct standard statistical tests. The next part of the SPSS workshop was conducted by Dr Datta, who demonstrated how to calculate correlation coefficients and perform regression analysis using a hypothetical set of data.

**Figure 5.**
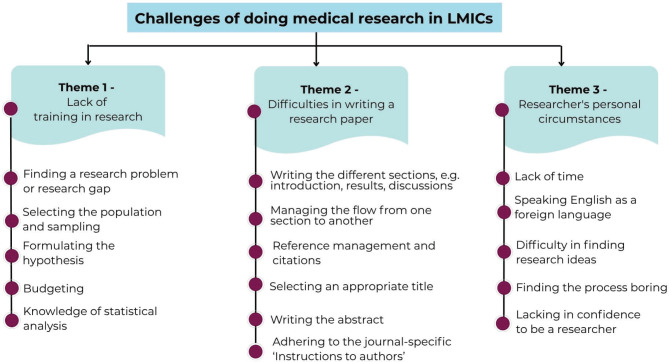
Synthesis of free text responses on challenges the participants face while writing a paper.

During the course, participants were asked via Mentimeter, ‘What’s the hardest part of writing a research paper?’ The obtained responses (*n* = 47) were analysed qualitatively, and the main themes that emerged are described in Figure 5. The major themes that emerged were a) Lack of training in research (‘finding a research problem or a gap’; ‘sampling participants’, ‘budgeting a project’), b) Difficulties in writing a research paper (‘organising different sections, ‘managing the flow from one section to another’, writing an abstract’ and so on) and c) Researcher’s personal circumstances (‘lack of time’, ‘anguage barriers with patient population’ and so on).

Finally, the closing session was conducted by Dr Rima Mukherjee, the current president of the West Bengal Chapter of the Indian Psychiatric Society. She thanked the audience and felicitated the organisers and faculty on behalf of the Indian Psychiatric Society.

#### Results of participant feedback

The ‘9th’ Tata Annual Biostatistics and Research Methodology course 2025 had a total of 94 participants, which included 81 delegates, ten faculty members and three organising committee members. 81 pre-registered delegates from various parts of India attended the course. Of these, 71 participants (71/81, 87%) provided written feedback. Of the 81 delegates, 44 were women (54%) and 37 were men (46%). There were 55 delegates (74%) who were early-career professionals, aged under 40 years. The programme attracted medical professionals (*n* = 55, 74%) from diverse specialities, along with participants from related disciplines (*n* = 26, 32%), including psychologists, medical students and allied health professionals.

**Figure 6.**
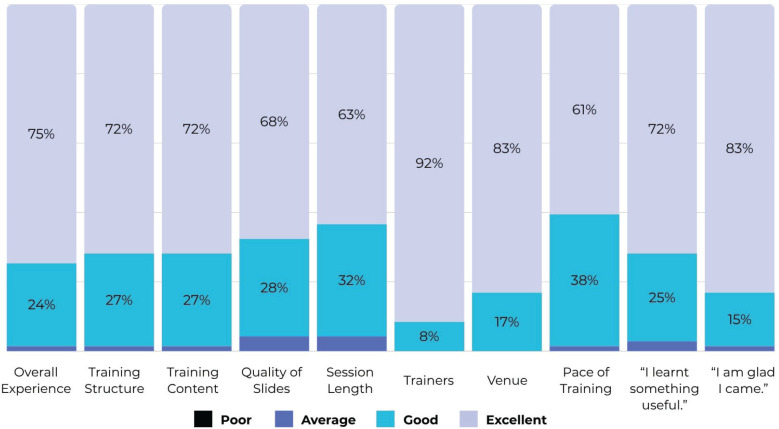
Quantitative ratings provided by participants (n = 71) at the end of the 2-day programme.

The majority of the feedback from the delegates was rated ‘excellent’, followed by ‘good’ in all aspects of the quantitative ratings, as shown in Figure 6. In the free-text part of the responses, they elaborated on specific aspects of the course they liked. The participants appreciated the hands-on training sessions on Zotero and SPSS.

The summary of free-text responses is presented in Table 1.

To summarise the above table (Table 1), the feedback has been grouped by the participants’ age, gender and current career standing. It was found that most participants appreciated the workshops on Zotero, PubMed search and Statistical analysis using SPSS and some requested longer sessions for these topics. They also enjoyed the multidisciplinary approach adopted by the trainers and the clear and concise delivery of the lectures.

Feedback was also collected in video format from the participants and faculty who consented to being recorded. They discussed the need to conduct a research methodology course, shared their key takeaways and reflected on the 2-day programme.

## Conclusion

Our findings suggest that post-graduate trainees in oncology and other specialities, qualified medical and nursing professionals working in oncology and medical academics were all eager to learn about research methods in a structured two-day course delivered by clinician-researchers. The underlying motivation for learning about medical research was varied, including improving existing knowledge, eagerness to design their own research studies, professional development and developing the capacity to apply emerging evidence in clinical practice. Published literature from other parts of the globe have similarly highlighted the positive impact of a research culture on patient experience, as well as the learning experience of trainees, resident doctors and the institutional culture [7]. The feedback identified gaps in knowledge about writing a research paper and a lack of understanding of strategies for responding to reviewers’ comments. This is not surprising, given the limited exposure to a research culture for the majority of delegates who participated in the research methodology course. The three main barriers to conducting research that our participants reported were ‘‘lack of training in research’, ‘difficulties in writing a research paper’ and the ‘researcher’s personal circumstances’. These findings corroborated with those reported from a survey conducted in 27 countries in Africa, which highlighted the barriers to clinical research and emphasised the lack of dedicated research teams in LMICs, paucity of staff skilled in research and poor team commitment to research in the face of high clinical load, alongside other resource-related issues and regulatory barriers [8]. After attending the course, participants mentioned in their feedback the value of hands-on training and appreciated the faculty's clear and straightforward approach to explaining complex concepts. Many participants requested frequent sessions and small group training.

In recent times, there has been significant discussion worldwide about the gender gap in scientific research and the under-representation of women in biomedical research teams and publications [9]. This is true for academic medicine, even in high-income countries as the US [10]. We are pleased to report that the majority of our participants were women in the early stages of their careers. One can hope that the gender gap in medical research will be closed over time as the female workforce in medicine increases worldwide and women become more inspired to pursue research as a career [11]. The government of India has initiated innovative programmes to encourage women in science under the umbrella of Women in Science and Engineering-KIRAN [12].

The majority of the global cancer burden is in LMICs [13], and more research is needed to originate from these areas. To achieve this, institutions and individuals need to foster a conducive environment for research, inspiring those who will be responsible for the future healthcare delivery.

## Conflicts of interest

The authors declared that they do not have any conflicts of interest.

## Funding

All faculties for the course taught pro-bono. Other organisational aspects were covered by participant registration fees and support from Tata Medical Center, Kolkata. The open-access fees for this project report was supported by ecancer Global Foundation.

## Figures and Tables

**Video 1.**
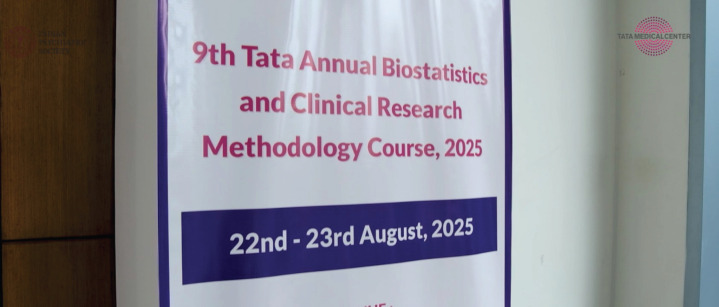
Overview of the 9th Tata Annual Biostatistics and Research Methods Course 2025 by Dr Soumitra Datta, Chair of Organising Committee, Dr Sanjay Garg, Organising Secretary, other faculty members and several participants. (See the video at https://vimeo.com/1166052267/f3b7037b0a?share=copy&fl=sv&fe=ci.)

**Video 2.**
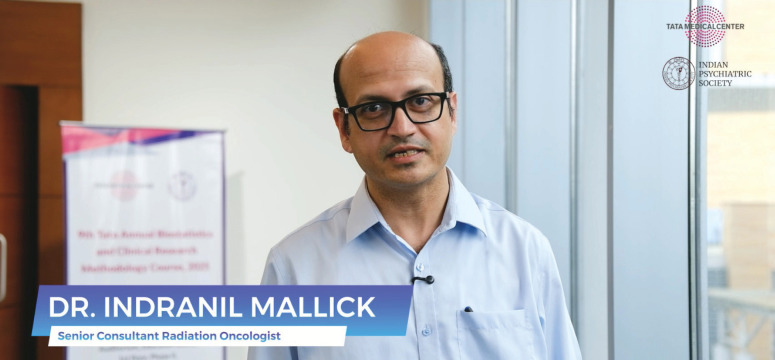
Reflections by Dr Indranil Mallick on the importance of conducting clinical research to impact patient care. (See the video at https://vimeo.com/1166048080/43f4beef14?share=copy&fl=sv&fe=ci.)

**Table 1.** Written feedback from participants after completion of the course.

Written feedback from participants			
Respondent(s)	What did you like about the course?	How could the course be improved?	Outline three things that you have learnt in this course
Early-career female participant(s)	‘The workshop helped me to make sense of many topics in research methodology and biostatistics’ (P15) ‘More practical-oriented than just theoretical jargon’ (P35) ‘Complex tools were explained step by step’ (P37)	‘A session on data collection can be included’ (P43) ‘More quiz questions may consolidate learning’ (P60) ‘Small group trainings may be useful’ (P29)	‘Learnt about research in resource-limited settings’ (P40)
Early-career male participant(s)	‘Adequate in hands-on learning’ (P1) ‘SPSS workshop was very useful’ (P8) ‘Learning to do reference management with Zotero’ (P13)	‘Training for more than 2 days would be helpful for beginners.’ (P10) ‘More teaching aids can be added’ (P4) ‘Small group (5–10 people) could be useful’ (P11) ‘Integrating AI in research can be covered in future’ (P62) ‘Add a session on bioinformatics’ (P24) ‘A session discussing plagiarism would be useful’ (P28)	‘Multi-disciplinary research work procedures were useful to learn’ (P46) ‘I will try to do my own stats in my projects’ (P24)
Mid-career female participant(s)	‘Relevant, crisp and plenty of information’ (P16) ‘I liked the session on logistic and linear regression’ (P23)	‘To include more practical examples’(P6) ‘If the workshop is done over multiple sessions over a few months, retention would improve.’ (P16)	‘I learnt using Zotero, PubMed search, and SPSS’ (P25) ‘I understood what statistical test to use in which situation’ (P45)
Mid-career male participant(s)	‘I liked the session on PubMed search techniques’ (P17) ‘The content is relevant for professional development’ (P71)	‘Pre-course manual and book can be used’ (P5) ‘A talk on how to account for missing data would be useful’ (P33) ‘I want to learn more about conducting a systematic review and meta-analysis’ (P38)	‘Learnt about troubleshooting in SPSS’ (P38) ‘Learnt about Zotero, PubMed search and Statistical analysis’ (P71)
